# Longitudinal Associations of Past and Cumulative Terrorism Exposure with Community Violence Exposure Among Israeli Adolescents: Aggression and Severe Violence Commission as Moderators

**DOI:** 10.3390/ijerph23081004

**Published:** 2026-07-31

**Authors:** Golan Shahar, Avi Besser, Christopher C. Henrich

**Affiliations:** 1 Department of Psychology, Ben-Gurion University of the Negev, P.O. Box 653, Beer-Sheva 8410501, Israel; 2 Department of Communication Disorders, Jerusalem Multidisciplinary College, Jerusalem 91010, Israel; 3 Department of Psychology, University of Alabama at Birmingham, 1300 University Boulevard, CH 415, Birmingham, AL 35233, USA; chenrich@uab.edu

**Keywords:** terrorism exposure, community violence exposure, adolescence, aggression, severe violence commission, dual vulnerability, rival vulnerability, Israel

## Abstract

**Highlights:**

**Public health relevance—How does this work relate to a public health issue?**

- Chronic terrorism exposure is a public health concern because its developmental consequences may extend beyond trauma-related distress to adolescents’ exposure to violence in their everyday social environments.
- This longitudinal study examines how past and cumulative terrorism exposure relate to community violence witnessing and victimization among Israeli adolescents living under chronic armed threat.

**Public health significance—Why is this work of significance to public health?**

- Associations between terrorism exposure and community violence exposure varied according to aggression and severe violence commission and differed between violence witnessing and violence victimization.
- The pattern was more consistent with dual-vulnerability or rival-vulnerability than with stress-diathesis: positive associations of past terror exposure with later violence witnessing emerged at low levels of aggression or severe violence commission and were attenuated or reversed at higher levels.

**Public health implications—What are the key implications or messages for practitioners, policy makers and/or researchers in public health?**

- Screening and prevention efforts in chronically threatened communities may benefit from jointly assessing terrorism exposure, aggression, severe violence commission, and community violence exposure.
- Public health responses may benefit from integrating trauma-informed adolescent mental health services with school- and community-based violence prevention while distinguishing violence witnessing from direct victimization.

**Abstract:**

Terrorism exposure may be longitudinally associated with adolescents’ community violence exposure, but these associations may depend on preexisting externalizing behavior. Guided by ecological–transactional theory and competing stress-diathesis, dual-vulnerability, and rival-vulnerability perspectives, this study examined whether aggression and severe violence commission moderated associations of past terror exposure and cumulative Kassam rocket exposure, referring to exposure to short-range rockets launched from the Gaza Strip toward southern Israel, with Time 2 violence witnessing and victimization. Participants were 362 Israeli adolescents enrolled in a longitudinal study with assessments in 2008 and 2010; cumulative Kassam rocket exposure incorporated exposure reports from 2008, 2009, and 2010. Models controlled for baseline community violence exposure, depressive and anxiety symptoms, sex, grade, and locality. Structural equation modeling (SEM) was used to test the hypothesized models. Under low levels of both aggression and severe violence commission, past terror exposure was prospectively associated with higher Time 2 violence witnessing. Under high levels of severe violence commission, past terror exposure was prospectively associated with lower Time 2 violence victimization. A three-way interaction involving past terror exposure, cumulative Kassam rocket exposure, and aggression was also found and interpreted with caution. Overall, the pattern was consistent with a dual-vulnerability or rival-vulnerability pattern, but not with a stress-diathesis model. This pattern highlights the importance of assessing terrorism exposure, externalizing behavior, and distinct forms of community violence exposure when designing preventive interventions for adolescents living under chronic armed threat.

## 1. Introduction

Exposure to terrorism constitutes a major developmental stressor for adolescents, particularly when it is chronic, recurrent, and embedded in daily life. During the study period, Sderot and neighboring communities in Shaar HaNegev were repeatedly exposed to attacks involving Kassam rockets, also commonly spelled Qassam, which are short-range rockets launched from the Gaza Strip toward southern Israel. The recurrence of these attacks over several years made them a chronic threat embedded in adolescents’ everyday lives at home, at school, and in the community [1,2]. Population-based and adolescent-focused studies conducted in Israel have shown that ongoing terrorism is associated with substantial mental health burden, including posttraumatic stress symptoms, functional impairment, depressive symptoms, suicidal ideation, and diminished sense of safety [1,2,3,4,5,6,7,8]. Prospective evidence from Sderot further indicates that exposure to rocket attacks predicts increases in adolescent depressive symptoms over time, particularly under conditions of low social support [1]. Together, these findings indicate that terrorism exposure in adolescence is associated not only with immediate fear and distress, but also with more sustained emotional, behavioral, and functional maladjustment.

### 1.1. Literature Review and Theoretical Framework

At the same time, the developmental consequences of terrorism exposure may extend beyond internalizing distress. Research conducted with Israeli adolescents suggests that recurrent or close physical exposure to terrorism is also associated with risk-taking, substance use, and violent behavior. Pat-Horenczyk et al. [6] found that recurrent terrorism exposure was associated with greater risk-taking behavior among Israeli adolescents. Similarly, Schiff et al. reported that close physical exposure to terrorism was linked to higher alcohol consumption, binge drinking, and cannabis use, even after controlling for posttraumatic stress and depressive symptoms [9]. Even-Chen and Itzhaky further showed that exposure to terrorism contributed uniquely to adolescents’ violent behavior above and beyond domestic and community violence and personal and environmental resources [10]. Most notably, Henrich and Shahar found in a four-year longitudinal study that repeated exposure to rocket attacks prospectively predicted violence commission, whereas long-term effects on anxiety and depressive symptoms were comparatively modest [2]. These findings suggest that terrorism exposure may be relevant not only to adolescents’ emotional distress, but also to their involvement in risky and violent behavior.

The possibility that terrorism exposure is also associated with externalizing problems becomes especially important when considered alongside the broader literature on community violence. Exposure to community violence is not only an indicator of environmental risk but also a predictor of later maladjustment. Gorman-Smith and Tolan, for example, found that community violence exposure predicted increases in aggressive behavior and depressive symptoms over time [11]. Gorman-Smith et al. further showed that exposure to community violence was associated with later violence commission, although this association was moderated by family functioning [12]. In Israeli early adolescents, Brookmeyer et al. found that witnessing community violence and witnessing terror violence jointly increased the risk of violent behavior, whereas support from parents, school, and peers functioned as protective factors [13]. Taken together, these findings suggest that terrorism exposure and community violence exposure may be developmentally linked within adolescents’ broader ecology of risk, rather than understood as wholly separate forms of adversity.

Prospective evidence from the broader violence-exposure literature further strengthens this developmental rationale. Mrug and Windle showed that exposure to violence across multiple contexts predicted later internalizing and externalizing problems among early adolescents, underscoring the importance of distinguishing among forms and contexts of violence exposure [14]. Farrell et al. further documented bidirectional relations between witnessing violence and physical aggression during adolescence, suggesting that aggression may both contribute to and result from continued involvement in violent environments [15]. Together, these findings support the possibility that chronic terrorism exposure may become linked over time to subsequent community violence exposure, and that preexisting aggression or severe violence commission may shape this developmental pathway.

A useful framework for understanding these links is the ecological–transactional model proposed by Cicchetti and Lynch. This model posits that child and adolescent development unfolds within nested ecological systems, and that broader social adversity may shape more proximal relational, family, peer, school, and community contexts [16]. Applied to the present study, chronic terrorism exposure may not only generate distress directly, but may also increase adolescents’ likelihood of becoming involved in unsafe peer, neighborhood, and community settings. Empirical work on political violence is broadly consistent with this perspective. Dubow et al. showed that exposure to political conflict retained unique associations with posttraumatic stress symptoms and aggressive behavior even when family, community, and school violence were considered simultaneously [17]. Similarly, Cummings et al. found that political violence predicted child maladjustment through family conflict and emotional insecurity [18]. Thus, both developmental theory and empirical work suggest that repeated terrorism exposure may plausibly contribute to adolescents’ later community violence exposure, including violence witnessing and violence victimization.

More recent longitudinal research has further clarified the emotional and social-cognitive processes through which persistent political violence may become linked to externalizing outcomes. Niwa et al. [19] identified developmental profiles characterized by high aggression and comparatively low emotional distress, consistent with emotional desensitization, and found that youths exhibiting such patterns were more likely to endorse aggression toward ethnic outgroups. Huesmann et al. [20] subsequently showed that exposure to ethnic–political violence predicted later aggression toward peers through changes in aggressive-script rehearsal, normative beliefs favoring aggression, and emotional distress. Extending this work across a longer developmental interval, Docherty et al. [21] found that cumulative violence exposure predicted later callous–unemotional traits and aggression, even after accounting for their earlier levels. Most recently, Boxer et al. [22] reported that chronic exposure across political, community, family, and school contexts predicted later aggression and posttraumatic stress, with persistent interethnic–political violence contributing uniquely to aggression. Together, these findings indicate that repeated political violence may shape later externalizing outcomes through both emotional attenuation and the social-cognitive normalization of aggression.

The key unresolved question, however, is whether this ecological effect is uniform across adolescents or whether it depends on adolescents’ preexisting aggression and severe violence commission. Diathesis-stress theory proposes that stressors may exert stronger effects among individuals who already possess relevant vulnerabilities [23]. Consistent with this broader developmental logic, Henrich and Shahar found that the prospective effect of peer victimization on later depressive and anxious–depressive symptoms was stronger among children who also engaged in bullying or showed higher baseline symptomatology [24]. In the present context, aggression and severe violence commission may reflect behavioral vulnerabilities that strengthen the longitudinal associations of past terror exposure and cumulative Kassam rocket exposure with Time 2 community violence exposure. This possibility is consistent with empirical findings showing that aggression is developmentally intertwined with violence exposure [15], and that repeated rocket exposure prospectively predicts violent behavior over time [2]. Accordingly, a diathesis-stress account predicts that these longitudinal associations will be stronger among adolescents with higher levels of aggression or severe violence commission.

At the same time, prior findings from Israeli adolescents suggest that a straightforward stress-diathesis account may not always hold. Henrich and Shahar showed that the psychological effects of terrorism exposure were conditional rather than uniformly linear [1]. More directly, Shahar and Henrich compared stress-diathesis and dual-vulnerability formulations in adolescents repeatedly exposed to rocket attacks [25]. Dual-vulnerability models suggest that two risk factors, such as stress and internal vulnerability, may compete in accounting for adverse outcomes: each risk factor exhibits an adverse effect primarily when levels of the other factor are low. Shahar and Henrich found this pattern for the adverse effects of adolescent personality vulnerability, specifically self-criticism, and cumulative exposure to Kassam rockets, and proposed that the term *rival-vulnerability* may better capture this dynamic than *dual-vulnerability* [25].

Although that study focused on personality vulnerability rather than aggression or severe violence commission, its findings are relevant to the present investigation because they suggest that preexisting vulnerability does not necessarily amplify the effects of terrorism exposure. Instead, vulnerability and environmental threat may sometimes compete in accounting for later maladjustment. Applied to the present context, this perspective raises the possibility that aggression and severe violence commission may not always strengthen the ecological effects of terrorism exposure and may, in some cases, attenuate its additional predictive contribution.

Distinguishing between aggression and severe violence commission is therefore theoretically important. These constructs are related but nonidentical forms of externalizing behavior. Aggression may reflect a relatively broad tendency toward interpersonal hostility, confrontation, and coercive behavior, whereas severe violence commission may index a more severe and behaviorally entrenched pattern of violent involvement. Existing empirical work suggests that terrorism exposure is linked to violent behavior in adolescents and that repeated rocket exposure prospectively predicts violence commission over time [2,10]. At the same time, broader developmental research suggests that aggression and violence exposure may be reciprocally linked over time [15]. Considered together, these findings justify examining whether aggression and severe violence commission differentially moderate the longitudinal associations of past terror exposure and cumulative Kassam rocket exposure with Time 2 community violence exposure.

### 1.2. The Present Study and Hypotheses

The present study tested whether past terror exposure and cumulative Kassam rocket exposure were longitudinally associated with Time 2 community violence exposure, operationalized as violence witnessing and violence victimization, and whether these associations were moderated by aggression and severe violence commission. Depressive symptoms and anxiety symptoms were included as covariates in order to account for broader internalizing psychopathology. This design allowed for evaluation of ecological–transactional, diathesis-stress, dual-vulnerability, and rival-vulnerability perspectives within a single longitudinal framework.

**H1.**  *Based on the ecological–transactional model, greater past terror exposure was expected to prospectively predict higher levels of Time 2 community violence exposure. Greater cumulative Kassam rocket exposure was likewise expected to be longitudinally associated with higher levels of Time 2 community violence exposure. These expectations applied to both violence witnessing and violence victimization*.

**H2a.**  *Based on the diathesis-stress model, the longitudinal associations of past terror exposure and cumulative Kassam rocket exposure with Time 2 community violence exposure were expected to be stronger at higher levels of aggression and severe violence commission [23]. More specifically, higher aggression and higher severe violence commission were expected to strengthen the positive associations of both forms of terrorism exposure with Time 2 community violence exposure*.

**H2b.**  *Conversely, based on the dual-vulnerability or rival-vulnerability model, elevated levels of aggression and severe violence commission were expected to attenuate or buffer the associations of past terror exposure and cumulative Kassam rocket exposure with Time 2 community violence exposure [25]*.

No distinct hypotheses were formulated regarding differences between aggression and severe violence commission or between the two outcomes, namely violence witnessing and violence victimization. In addition, interactions among aggression or severe violence commission, past terror exposure, and cumulative Kassam rocket exposure were examined on an exploratory basis.

## 2. Materials and Methods

### 2.1. Participants and Study Procedure

The present study drew on data from a longitudinal sample of adolescents residing in the Northern Negev region of Israel, including Sderot and Shaar HaNegev, an area characterized during the study period by chronic exposure to terrorism and Kassam rocket attacks. The present analyses focused on self-report assessments conducted in 2008 (Time 1) and 2010 (Time 2), while also incorporating data on Kassam rocket exposure collected at the intermediate 2009 assessment. The original project included four annual waves of data collection (2008, 2009, 2010, and 2011); however, the 2011 wave was excluded from the present analyses because it included only participants from Shaar HaNegev (see [25]). Accordingly, cumulative Kassam rocket exposure was calculated using data from the 2008, 2009, and 2010 assessments.

Participants were recruited through schools in Sderot and Shaar HaNegev. Parents or legal guardians received written information about the study and were given ten days to decline their child’s participation. Adolescents whose parents or legal guardians did not object and who themselves assented participated in the study. Adolescents completed the assessment battery in school settings under the supervision of trained research assistants. Participants were informed that their responses would remain confidential and would be used for research purposes only. All pilot work and data collection began only after the required ethical and governmental approvals had been obtained. The project was reviewed and conditionally approved by the Ethics Committee of Ben-Gurion University of the Negev on 31 December 2006, subject to authorization by the Chief Scientist of the Israeli Ministry of Education. The required authorization from the Chief Scientist was obtained in 2007, before any pilot work or school-based data collection began. The surviving archival document is a continuation authorization for a later phase of the project, issued under reference no. 10.32.953 on 7 September 2009. The collaborative project was also approved by the Institutional Review Board of Georgia State University, where the U.S. principal investigator was affiliated at the time, before the relevant research activities were conducted. Because the project began nearly two decades ago, the original protocol codes and archival documents for the Ben-Gurion University and Georgia State University approvals, as well as the code and exact date of the initial 2007 Chief Scientist authorization, are no longer retrievable. No adverse events were documented. The present secondary analysis of de-identified archival data was reviewed separately by the Institutional Review Board of the University of Alabama at Birmingham. On 15 July 2026, the UAB IRB determined that the project, “Secondary Analysis of the Development Under Duress Study,” did not constitute human subject research (IRB-300017092).

Details of the demographic makeup of the sample are reported in Shahar and Henrich [25]. Briefly, participants were 362 Israeli adolescents in Grades 7–10, 54% of whom were female. Most participants were born in Israel (89.7%), and the demographic composition of the sample mirrored that of the student bodies of the two participating schools.

As reported by Shahar and Henrich [25], attrition resulted from participants moving away, progressing beyond the participating school grades, entering military service, or being unable to participate because of security-related restrictions. Of the full longitudinal study cohort of 362 adolescents, 37 participants (10.2%) were missing data from the 2008 assessment, 102 participants (28.2%) were missing data from the 2010 assessment, and 130 participants (35.9%) were missing some data across the focal assessment waves. At the intermediate 2009 assessment, data were available for 305 adolescents, including 31 who had not participated in the 2008 assessment. Little’s test of missing completely at random was nonsignificant, χ^2^(170) = 178.39, *p* = 0.31, providing some evidence consistent with MCAR. However, a logistic regression analysis reported by Shahar and Henrich [25] showed that participants from Sderot were more likely than those from Shaar HaNegev to be missing at the 2010 assessment, with OR = 2.57, 95% CI [1.47, 4.49], *p* = 0.001. Accordingly, although Little’s test did not reject the MCAR hypothesis, the missingness pattern may be more appropriately regarded as plausibly missing at random conditional on observed locality rather than as unequivocally MCAR. Locality was therefore included as a covariate in all primary models, and missing data were handled using full-information maximum likelihood.

### 2.2. Materials

Study variables were assessed by adolescent self-report questionnaires administered in 2008 and 2010, with additional data on Kassam rocket exposure collected in 2009 for the construction of the cumulative exposure measure, as detailed below. The focal predictors were past terror exposure and cumulative Kassam rocket exposure across the study interval. The focal moderators were aggression and severe violence commission. Depressive symptoms and anxiety symptoms were included as covariates, and the primary outcomes were Time 2 community violence witnessing and Time 2 community violence victimization. Sex, locality, grade, and baseline violence exposure were also statistically controlled.

#### 2.2.1. Past Terror Exposure

Past terror exposure was assessed at Time 1 using a five-item yes/no measure assessing whether adolescents had ever been present during a terror or rocket attack, had themselves been injured in such an attack, had a close family member been injured, had a close friend been injured, or had acquaintances been injured [2]. Item responses were summed to create a composite score ranging from 0 to 5, with higher scores indicating greater prior exposure to terrorism. This variable served as one of the two focal terrorism-exposure predictors in the longitudinal models. Because these items represent distinct and noninterchangeable exposure events whose occurrence is not expected to covary, the measure was treated as a formative event-count index, and internal consistency was not estimated.

#### 2.2.2. Cumulative Kassam Rocket Exposure

Cumulative Kassam rocket exposure during the study interval was assessed using a six-item exposure checklist previously used with adolescents in southern Israel exposed to rocket attacks [2]. At each of the 2008, 2009, and 2010 assessments, adolescents were asked six yes/no questions regarding whether, during the past several months, they had been physically hurt in a rocket attack, had experienced property damage from a rocket attack, or had friends or family members who were physically or psychologically harmed or whose property was damaged in a rocket attack. Item responses were summed at each assessment to yield a wave-specific exposure score ranging from 0 to 6, with higher scores indicating greater exposure to Kassam rocket attacks. For the present study, cumulative Kassam rocket exposure was operationalized as the sum of the three wave-specific exposure scores, yielding a possible range of 0 to 18, with higher values reflecting greater cumulative exposure to Kassam rocket attacks across the study interval. Because the checklist assesses distinct and noninterchangeable exposure events rather than reflective indicators of a unitary latent construct, internal consistency was not estimated.

The 2010 assessment was retained in this cumulative measure to capture the adolescents’ total Kassam rocket exposure across the full study interval and to maintain continuity with the operationalization used in previous analyses of this longitudinal project [2,25]. However, because the 2010 exposure assessment occurred during the same assessment year as the Time 2 community violence outcomes, associations involving cumulative Kassam rocket exposure are interpreted as longitudinal associations across the study interval rather than as strictly prospective effects.

#### 2.2.3. Depressive Symptoms

Depressive symptoms were assessed at Time 1 using the Center for Epidemiological Studies Depression Scale for Children (CES-DC; [26]), a 20-item self-report measure of depressive symptoms. Item scores were summed to create a composite score, with higher values indicating greater depressive symptom severity. Internal consistency at Time 1 was acceptable (α = 0.81). In the present study, depressive symptoms were included as a covariate to account for broader internalizing psychopathology.

#### 2.2.4. Anxiety Symptoms

Anxiety symptoms were assessed at Time 1 using a seven-item version of the Hebrew State Anxiety Inventory (SAI; [27,28]). A sample item is “I am anxious.” Items were averaged to create a composite score, with higher values indicating greater anxiety symptom severity. Internal consistency at Time 1 was good (α = 0.85). In the present study, anxiety symptoms were included as a covariate to account for broader internalizing psychopathology.

#### 2.2.5. Aggression

Aggression was assessed at Time 1 using the 11-item Aggression Scale [29], a self-report measure of aggressive behavior in the past week. The scale assesses behaviors such as hitting, pushing, name calling, and threatening. Items were rated on a 0-to-6 frequency scale and averaged to create a composite score, with higher values indicating greater aggression. Internal consistency at Time 1 was excellent (α = 0.90). In the present study, aggression was examined as a moderator of the longitudinal associations of past terror exposure and cumulative Kassam rocket exposure with Time 2 community violence exposure.

#### 2.2.6. Severe Violence Commission

Severe violence commission was assessed at Time 1 using a four-item index derived from the Social and Health Assessment (SAHA; [30]). The index assessed adolescents’ involvement in severe violent behaviors during the past year, including seriously injuring someone in a physical fight, participating in a gang fight, being arrested for a violent crime, and carrying a weapon. Items were coded 0 (no) or 1 (yes) and summed to create a count score ranging from 0 to 4, with higher scores indicating involvement in a greater number of severe violent acts. In the present study, severe violence commission was examined as a moderator of the longitudinal associations of past terror exposure and cumulative Kassam rocket exposure with Time 2 community violence exposure. Because the items represent distinct severe violent acts whose occurrence is not necessarily expected to covary, the measure was treated as a formative behavioral count index, and internal consistency was not estimated.

#### 2.2.7. Community Violence Exposure: Witnessing and Victimization

Community violence witnessing and victimization were assessed using self-report measures adapted from Richters and Martinez [31], with shortened forms that have demonstrated reliability in this age group in prior research (e.g., [13,30,32]). Community violence witnessing assessed the degree to which adolescents had witnessed different forms of violent behavior in their neighborhoods during the past year. The witnessing scale consisted of six items. Each item was coded 0 if the event had never occurred and 1 if it had occurred one or more times, and the items were summed to create a score ranging from 0 to 6, with higher scores indicating exposure to a greater number of witnessed violence events. Adolescents were asked whether they had “seen the following things happen in your neighborhood in the past year.” Internal consistency was acceptable at both assessments (α = 0.73 at Time 1 and α = 0.72 at Time 2).

Community violence victimization assessed the degree to which adolescents had personally experienced different forms of community violence during the past year. The victimization scale also consisted of six items. Each item was coded 0 if the event had never been experienced and 1 if it had occurred one or more times, and the items were summed to create a score ranging from 0 to 6, with higher scores indicating victimization by a greater number of violence events. Internal consistency was acceptable at both assessments (α = 0.68 at Time 1 and α = 0.76 at Time 2).

#### 2.2.8. Covariates

Sex, locality, and grade served as demographic covariates. In addition, baseline community violence exposure variables were included to account for continuity in violence exposure across time. Specifically, Time 1 violence witnessing and Time 1 violence victimization were included as covariates when predicting the corresponding Time 2 outcomes. Depressive symptoms and anxiety symptoms were also included as covariates in all primary models to account for broader internalizing psychopathology.

### 2.3. Analytic Strategy

To maintain continuity with previous analyses [25], we used structural equation modeling with the full longitudinal study cohort (*n* = 362) to test the study hypotheses. Analyses were conducted in IBM SPSS AMOS (version 29) [33] using conventional maximum-likelihood estimation with full-information maximum likelihood (FIML) for missing data [33]. Amos does not implement the MLR or WLSMV robust estimators. Accordingly, FIML was used to retain all available information under missingness, but no estimator specifically providing robust standard errors for severe non-normality was applied. In Stage 1, model-implied means, variances, standard deviations, and zero-order intercorrelations were estimated for all study variables. In Stage 2, a series of observed-variable structural equation models were estimated to test the study hypotheses. The primary models included two Time 2 outcomes: violence witnessing and violence victimization. The residuals of these two outcomes were allowed to correlate. Three sets of models were run. In an initial, main effects model, the Time 2 outcomes were regressed on sex, grade, locality, Time 1 violence witnessing, Time 1 violence victimization, past terror exposure, cumulative Kassam rocket exposure, depressive symptoms, anxiety symptoms, aggression, and severe violence commission. Depressive symptoms and anxiety symptoms were included as covariates to account for broader internalizing psychopathology.

In the second set of models, testing moderation effects, two-way interactions of either aggression or severe violence commission with past terror exposure, and cumulative Kassam rocket exposure were examined separately (i.e., one model for aggression, and a second model for severe violence commission). The third set of models added the corresponding three-way interaction term (aggression × past terror exposure × Kassam exposure in one model, and severe violence commission × past terror exposure × Kassam exposure in a second model). All variables included in interaction terms were standardized to facilitate interpretation. Because the SEMs were saturated models, global fit indices were not informative. Significant interactions were probed by examining conditional effects at low and high levels of the relevant conditioning variables, defined as 1 SD below and 1 SD above the mean. The aggression and severe violence commission moderation models were estimated separately in order to limit the number of interaction parameters included within any single model. The three-way interactions were examined on an exploratory basis. Because the present study involved a secondary analysis of a fixed historical longitudinal cohort, the sample size was not determined through an a priori power analysis specifically designed for higher-order interaction effects. Accordingly, three-way interaction findings were interpreted cautiously.

## 3. Results

### 3.1. Stage 1: Descriptive Statistics

Table 1 presents means, variances, and standard deviations for all continuous study variables included in the main models.

Table 2 presents the zero-order intercorrelations among all study variables included in the main models. As shown, past terror exposure was positively associated with cumulative Kassam rocket exposure, depressive symptoms, anxiety symptoms, aggression, and both baseline and later violence witnessing. In addition, aggression and severe violence commission were positively associated with both forms of later community violence exposure, and baseline violence witnessing and victimization were positively associated with their corresponding Time 2 outcomes. These patterns supported the use of multivariate models to examine the unique and interactive longitudinal associations of past terror exposure, cumulative Kassam rocket exposure, aggression, and severe violence commission with Time 2 community violence exposure.

### 3.2. Stage 2: SEMs

Following these preliminary analyses, hierarchical observed-variable structural equation models were used to test the study hypotheses.

#### 3.2.1. Main Effects Model

The main effects model accounted for 25.3% of the variance in Time 2 violence witnessing and 13.1% of the variance in Time 2 violence victimization. The residual correlation between the two Time 2 outcomes was significant (r = 0.32, *p* < 0.001). Time 1 violence witnessing was the only statistically significant predictor of Time 2 violence witnessing (b = 0.300, SE = 0.071, t = 4.231, β = 0.331, *p* < 0.001). Two variables significantly predicted Time 2 violence victimization: Time 1 violence victimization (b = 0.181, SE = 0.087, t = 2.026, β = 0.145, *p* < 0.05) and past terror exposure (b = −0.087, SE = 0.043, t = −2.081, β = −0.184, *p* < 0.05). The negative adjusted association for past terror exposure should be interpreted cautiously, particularly because the corresponding zero-order correlation with Time 2 violence victimization was close to zero. This pattern suggests a possible suppressor effect and is revisited in the moderation models below.

#### 3.2.2. Aggression as a Focal Moderator

Model 2, which added two-way interactions, accounted for 35.6% of the variance in Time 2 violence witnessing and 19.9% of the variance in Time 2 violence victimization. The interaction between past terror exposure and aggression significantly predicted Time 2 violence witnessing (b = −0.472, SE = 0.090, t = −5.266, β = −0.600, *p* < 0.001). This interaction also significantly predicted Time 2 violence victimization (b = −0.217, SE = 0.055, t = −3.911, β = −0.552, *p* < 0.001). In addition, the interaction between cumulative Kassam rocket exposure and aggression significantly predicted Time 2 violence victimization (b = 0.133, SE = 0.053, t = 2.500, β = 0.340, *p* < 0.05).

Model 3, which added the three-way interaction, accounted for 34.5% of the variance in Time 2 violence victimization, whereas the explained variance in Time 2 violence witnessing remained unchanged. The three-way interaction among aggression, past terror exposure, and cumulative Kassam rocket exposure significantly predicted Time 2 violence victimization (b = −0.077, SE = 0.037, t = −2.100, β = −0.329, *p* < 0.05). Thus, the two-way interactions predicting Time 2 violence victimization were qualified by the three-way interaction. In contrast, the interaction between aggression and past terror exposure predicting Time 2 violence witnessing was not qualified by a three-way interaction.

**Figure 1:**
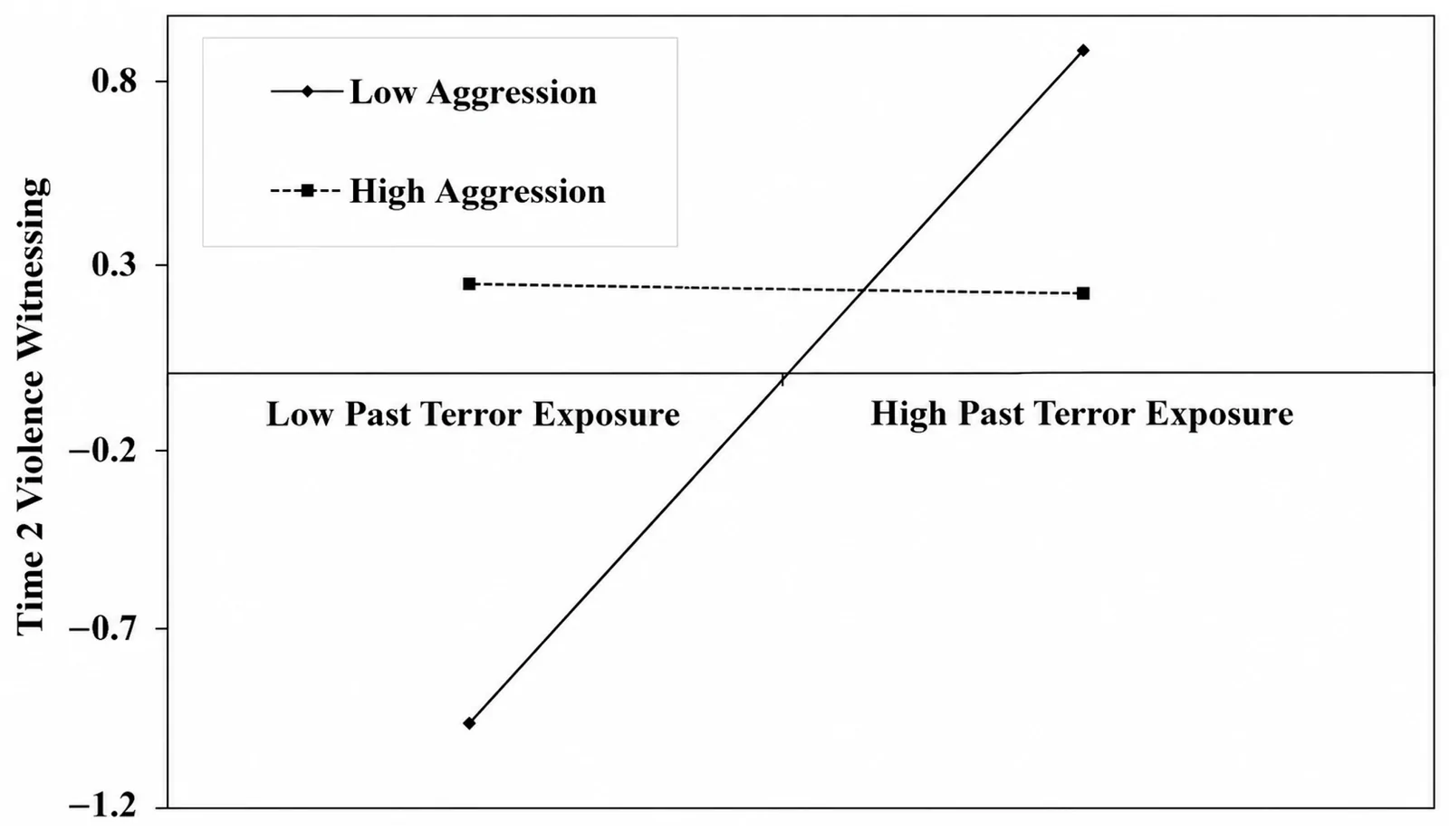
Time 2 violence witnessing as a function of Time 1 past terror exposure and Time 1 aggression. Note. Under low levels of aggression, higher past terror exposure was associated with higher Time 2 violence witnessing. Under high levels of aggression, this association was attenuated. Predicted values are adjusted for covariates and baseline violence exposure.

Simple-slope analyses were conducted to clarify the significant interactions from Models 2 and 3. First, the conditional effect of past terror exposure on Time 2 violence witnessing was examined at low and high levels of aggression, defined as 1 SD below and 1 SD above the mean. Results of this two-way interaction are presented in Figure 1. Past terror exposure significantly predicted higher Time 2 violence witnessing when aggression was low (b = 0.932, SE = 0.210, t = 4.446, β = 0.636, *p* < 0.001). Under high levels of aggression, this association was nonsignificant.

Probing the three-way interaction predicting Time 2 violence victimization required examining the conditional effect of past terror exposure across the four combinations of aggression and cumulative Kassam rocket exposure. Past terror exposure significantly predicted higher Time 2 violence victimization only when Time 1 aggression was low and cumulative Kassam rocket exposure was high (b = 0.417, SE = 0.136, t = 3.071, β = 0.574, *p* = 0.002).

The conditional effect of cumulative Kassam rocket exposure on Time 2 violence victimization was also examined across combinations of aggression and past terror exposure. The only significant conditional effect emerged when both past terror exposure and aggression were low. Under these conditions, cumulative Kassam rocket exposure was negatively associated with Time 2 violence victimization (b = −0.550, SE = 0.189, t = −2.946, β = −0.760, *p* < 0.01).

#### 3.2.3. Severe Violence Commission as a Focal Moderator

**Figure 2:**
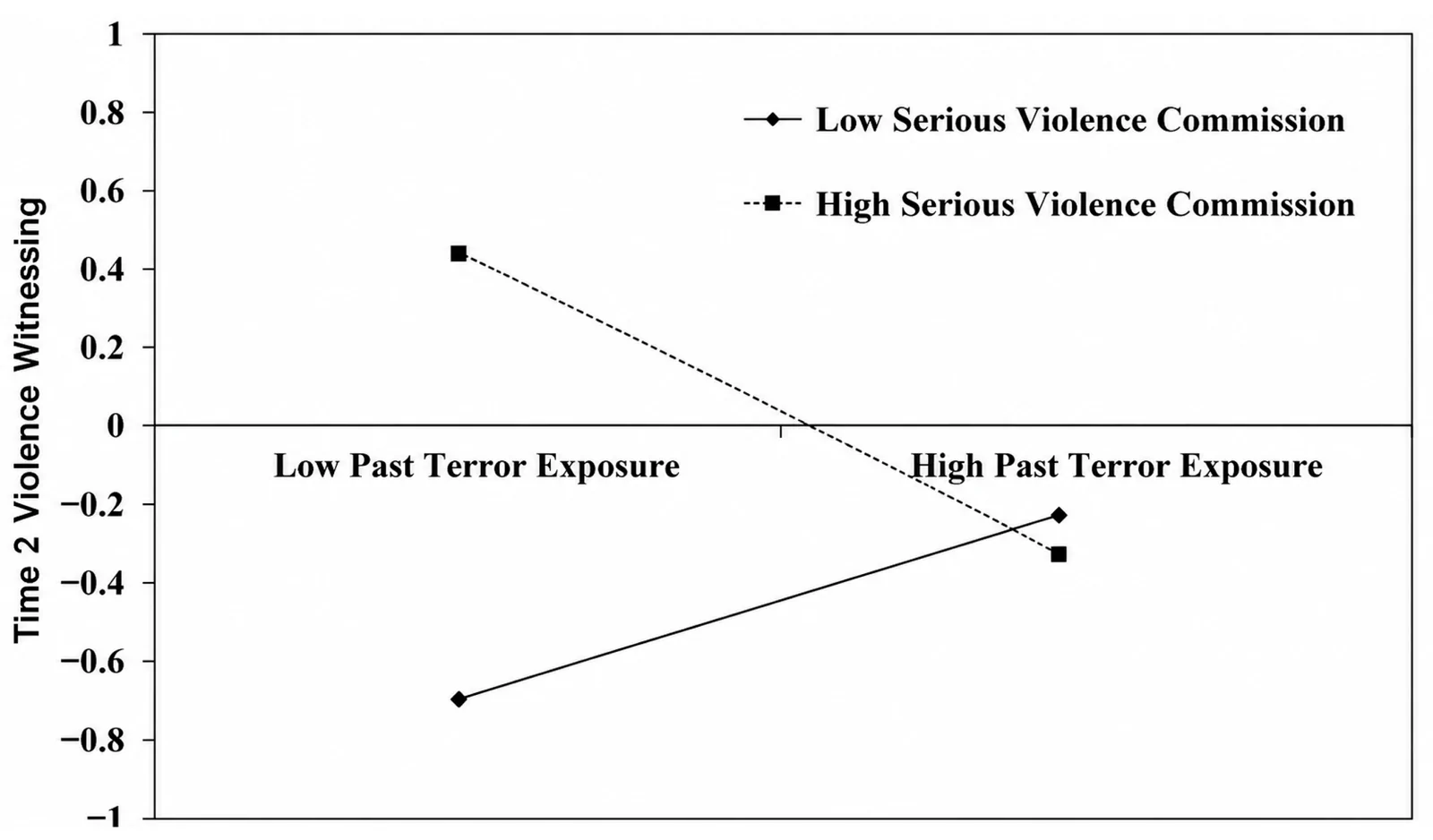
Time 2 violence witnessing as a function of Time 1 past terror exposure and Time 1 severe violence commission. Note. Under low levels of severe violence commission, higher past terror exposure was associated with higher Time 2 violence witnessing. Under high levels of severe violence commission, this association was reversed, such that higher past terror exposure was associated with lower Time 2 violence witnessing. Predicted values are adjusted for covariates and baseline violence exposure.

**Figure 3:**
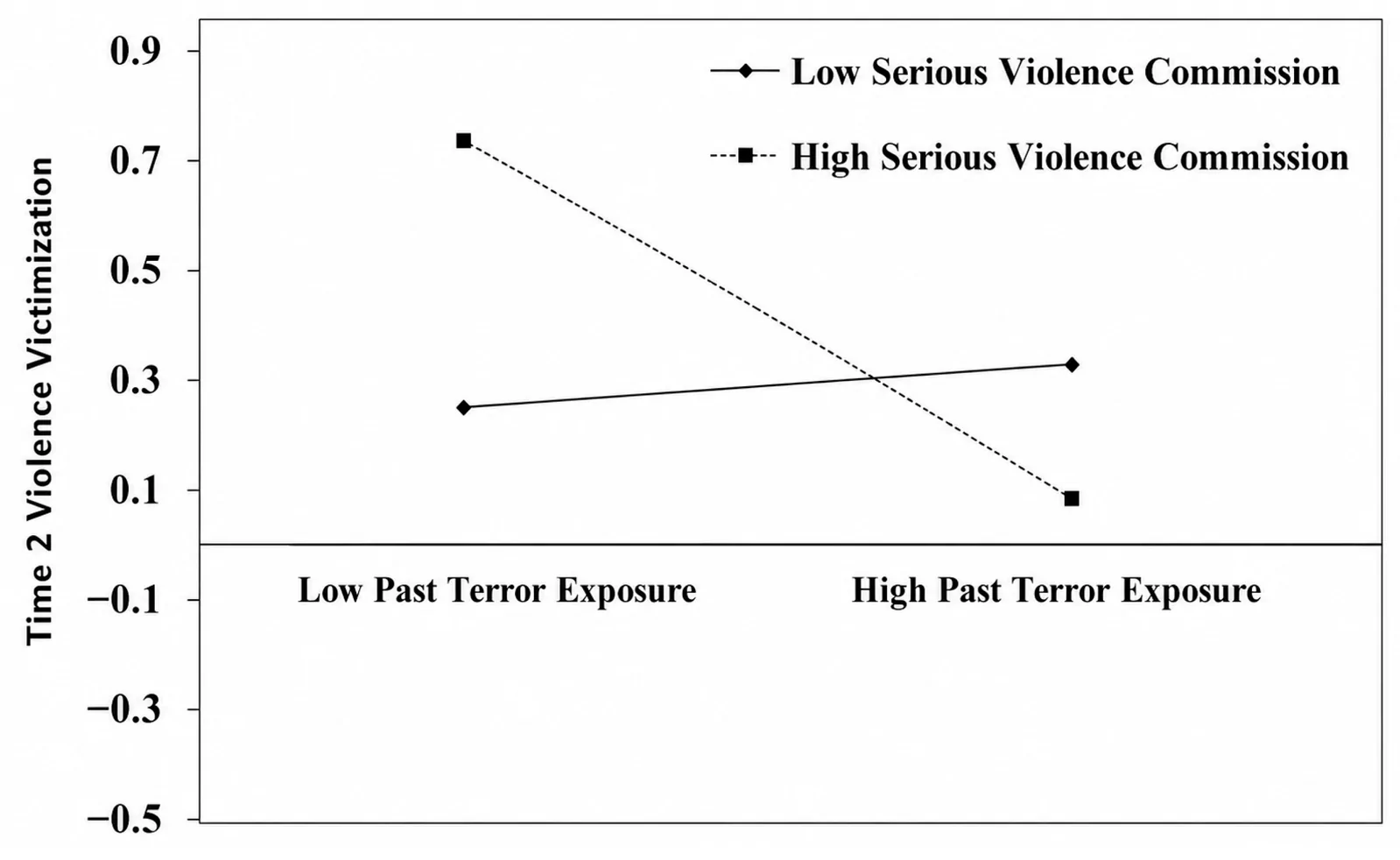
Time 2 violence victimization as a function of Time 1 past terror exposure and Time 1 severe violence commission. Note. Under higher levels of severe violence commission, higher past terror exposure was associated with lower Time 2 violence victimization. Under low levels of severe violence commission, the association was weak and nonsignificant. Predicted values are adjusted for covariates and baseline violence exposure.

In Block 2, which added the two-way interactions involving severe violence commission, the model accounted for 29.9% of the variance in Time 2 violence witnessing and 27.9% of the variance in Time 2 violence victimization. The interaction between past terror exposure and severe violence commission significantly predicted both Time 2 violence witnessing (b = −0.296, SE = 0.106, t = −2.930, β = −0.268, *p* < 0.01) and Time 2 violence victimization (b = −0.164, SE = 0.063, t = −2.610, β = −0.288, *p* < 0.01). As shown in Figure 2 and Figure 3, when severe violence commission was high, past terror exposure was negatively associated with both violence witnessing (b = −0.510, SE = 0.146, t = −3.484, β = −0.356, *p* < 0.001) and violence victimization (b = −0.364, SE = 0.084, t = −4.305, β = −0.470, *p* < 0.001). When severe violence commission was low, past terror exposure was positively associated with violence witnessing (b = 0.398, SE = 0.141, t = 2.825, β = 0.277, *p* < 0.01), whereas the corresponding association with violence victimization was nonsignificant. The three-way interaction in Block 3 was nonsignificant.

## 4. Discussion

The present study examined the longitudinal associations of past terror exposure and cumulative Kassam rocket exposure with Time 2 community violence exposure among Israeli adolescents living under chronic threat, and whether these associations were conditioned by aggression and severe violence commission. Overall, the findings suggest that terrorism exposure and community violence exposure may be developmentally linked, but that this linkage is not uniform across adolescents or across forms of violence exposure. Rather, the longitudinal associations varied according to the form of terrorism-related exposure, the form of externalizing behavior, and the outcome under consideration. The clearest and most consistent pattern emerged for violence witnessing.

The negative coefficient linking past terror exposure with Time 2 violence victimization in the main-effects model also warrants careful interpretation. Because the corresponding zero-order association was nonsignificant, the negative multivariate coefficient appears to reflect a suppressor or net-effect pattern that emerged only after accounting for baseline community violence exposure, aggression, severe violence commission, and internalizing symptoms. These covariates capture overlapping dimensions of behavioral and contextual risk that are positively associated with later victimization. Once this shared risk variance was statistically controlled, the residual component of past terror exposure may have reflected processes associated with reduced exposure to situations in which direct victimization could occur. Possible mechanisms include heightened vigilance, avoidance of public or high-risk settings, increased parental monitoring, or school and community restrictions during periods of threat. However, none of these processes was measured directly, and the finding should not be interpreted as evidence that terrorism exposure is protective. The pattern may reflect behavioral adaptation, statistical suppression, or both, and its underlying mechanism should be examined directly in future research.

Importantly, the findings do not support a broad stress-diathesis account in which aggression or severe violence commission consistently amplify the associations of terrorism exposure with later community violence exposure [23]. Instead, the observed interaction pattern was more consistent with a dual-vulnerability or rival-vulnerability framework, particularly for past terror exposure [25]. Empirically, the models showed that the conditional association between past terror exposure and later community violence exposure was attenuated, and in some cases reversed, at higher levels of externalizing behavior. These findings suggest a differential moderation pattern but do not identify the mechanism responsible for it.

One possible interpretation is a “no room” account, according to which externalizing behavior already captures substantial risk-related variance, leaving limited additional predictive variance for past terror exposure. A second, more speculative, possibility is a risky-resilience process, in which one risk-related process partly offsets another [25,26]. However, the study did not assess the attentional, behavioral, interpersonal, monitoring, or intervention-related processes required to distinguish between these explanations. The findings should therefore be understood as supporting a conditional association pattern, rather than as confirming a specific psychological mechanism.

One striking feature of the present findings is that the clearest and most consistent effects emerged for violence witnessing rather than violence victimization. This discrepancy likely reflects both a statistical constraint and a substantive developmental distinction. The relatively low base rate and restricted variance of direct victimization may have limited the ability to detect stronger associations with this outcome. At the same time, witnessing violence may be a particularly sensitive indicator of adolescents’ increasing exposure to unsafe social contexts, because it can rise as youths spend more time in risky peer, school, neighborhood, or community settings even when they are not directly targeted. Victimization, by contrast, requires a specific interpersonal encounter in which the adolescent becomes the target and may therefore depend more strongly on situational contingencies, confrontation, and opportunity. Adolescents may thus become increasingly immersed in unsafe environments and witness more violence without necessarily experiencing a parallel increase in direct victimization.

This interpretation is broadly consistent with longitudinal evidence showing that different forms and contexts of violence exposure do not contribute uniformly to later adjustment and that witnessing violence may have distinct developmental implications [14,15]. The stronger witnessing effects observed here therefore suggest that terrorism exposure may be linked more clearly to adolescents’ later proximity to unsafe social contexts than to direct victimization per se.

Cumulative Kassam rocket exposure was implicated only in the three-way interaction involving cumulative Kassam rocket exposure, past terror exposure, and aggression in the prediction of violence victimization. This finding requires an important temporal qualification. Because the cumulative exposure measure incorporated reports from 2008, 2009, and 2010, and the violence outcomes were also assessed in 2010, associations involving cumulative Kassam rocket exposure cannot be interpreted as strictly prospective or causal. The measure represents accumulated exposure across the study interval, but its 2010 component was contemporaneous with the Time 2 outcomes.

Within this qualified interpretation, high cumulative Kassam rocket exposure strengthened the positive association between past terror exposure and violence victimization when aggression was low. Elevated aggression again appeared to attenuate this conditional association, consistent with a rival-vulnerability pattern. Interestingly, when both aggression and past terror exposure were low, cumulative Kassam rocket exposure was negatively associated with violence victimization. This negative conditional association should not be interpreted as evidence that cumulative Kassam rocket exposure was protective. Rather, it suggests that the meaning of cumulative exposure may depend on the broader configuration of prior terrorism exposure and externalizing behavior.

Because this three-way interaction was exploratory and was estimated in a moderate sample using variables with restricted or skewed distributions, it should be regarded as tentative and hypothesis-generating. It does not provide definitive evidence of a specific triple-vulnerability process, and independent replication in larger conflict-exposed samples is required. Future longitudinal research should distinguish between distal and ongoing terrorism-related exposure, between broad aggression and severe violence commission, and between violence witnessing and violence victimization. These distinctions may help clarify when terrorism exposure functions as a developmental risk factor, when its association with later community violence exposure is attenuated in the context of preexisting externalizing behavior, and when different forms of threat and behavioral risk jointly shape adolescents’ subsequent exposure to community violence.

It is also important to clarify what the present findings do and do not imply about internalizing psychopathology. Depressive symptoms and anxiety symptoms were statistically controlled throughout the analyses in order to reduce the likelihood that the observed moderation effects merely reflected broader internalizing distress. Accordingly, the present findings do not suggest that internalizing symptoms are unimportant. On the contrary, earlier work has clearly shown that terrorism exposure is associated with depressive symptoms, anxiety, posttraumatic distress, and functional impairment among Israeli adolescents [1,4,5,6]. Rather, the present results suggest that, even after accounting for those internalizing dimensions, aggression and severe violence commission continue to shape the prospective associations of past terror exposure and the longitudinal study-interval associations of cumulative Kassam rocket exposure with Time 2 community violence exposure. In that sense, the present study refines, rather than replaces, the internalizing account.

### 4.1. Clinical Implications

The present findings have several clinical implications. First, adolescents exposed to chronic terrorism may warrant assessment not only for trauma-related distress, depressive symptoms, and anxiety symptoms, but also for later violence exposure, violent behavior, and broader contextual risk. Prior Israeli studies have shown that recurrent terrorism exposure is associated with functional impairment, risk-taking behavior, substance use, and violent behavior among adolescents [4,5,6,9,10]. The present findings suggest that Time 2 community violence exposure may also represent a clinically relevant domain of assessment, particularly in the form of violence witnessing.

Second, intervention planning may benefit from distinguishing between aggression and severe violence commission. Adolescents high in aggression may benefit from interventions targeting coercive interpersonal patterns, impulsive confrontation, emotion regulation, and risky peer or school contexts. This implication is consistent with developmental evidence showing reciprocal links between aggression and violence exposure [15]. By contrast, adolescents high in severe violence commission may warrant more intensive, behaviorally focused, multisystemic intervention approaches targeting violent behavior and unsafe social environments. The present findings suggest that this subgroup should not simply be understood as more vulnerable in a linear stress-diathesis sense; rather, severe violence commission may indicate an already entrenched risk pathway that changes the developmental meaning of prior terrorism exposure.

More broadly, clinical assessment and intervention in chronically threatened communities may benefit from extending beyond trauma-related symptoms alone. Trauma-focused assessment remains essential, but it may be insufficient when adolescents are also exposed to violent peer, school, neighborhood, or community contexts. Clinicians and school-based professionals may therefore consider assessing terrorism exposure, internalizing symptoms, aggression, severe violence commission, and community violence exposure as interconnected domains of risk. School-based interventions in conflict-exposed communities may therefore require a dual focus. Alongside screening and treatment for posttraumatic stress, anxiety, and depressive symptoms, programs may need to address the possible normalization of aggressive behavior and repeated exposure to violence. Relevant components could include perspective taking, empathy-promoting activities, emotion regulation, nonviolent conflict resolution, restorative peer practices, and skills for responding safely when violence is witnessed. These components may be particularly important for adolescents who are repeatedly exposed to violence in peer, school, neighborhood, or community settings, even when they are not direct victims. At the systems level, integration of trauma-informed mental health services with school-based violence prevention may warrant consideration with school-based violence prevention and social–emotional learning rather than treating political violence exposure and community violence as unrelated domains. Because desensitization, empathy, and conflict-resolution skills were not directly assessed in the present study, these recommendations should be regarded as theoretically informed clinical implications rather than as intervention effects demonstrated by the current findings.

### 4.2. Public Health Implications

The public health relevance of the present findings lies in the possibility that chronic terrorism exposure may be associated with violence-related risks extending beyond trauma-related distress. Chronic armed threat may therefore be usefully considered not only in relation to psychiatric symptoms, but also in relation to youth safety, community violence exposure, and broader developmental functioning. This interpretation is consistent with evidence from a nationally representative Israeli sample showing associations of ongoing terrorism with stress-related symptoms, probable posttraumatic stress disorder, and reduced sense of safety [3], as well as review evidence documenting multidomain consequences of war, terrorism, and armed conflict among children [34]. However, the present findings concern a specific adolescent sample and should not be understood as demonstrating a general causal progression from terrorism exposure to community violence exposure.

From a public health perspective, the findings are consistent with the potential value of approaches that consider trauma-related distress, adolescent mental health, externalizing behavior, and exposure to community violence together in chronically threatened areas. Screening and prevention efforts may benefit from considering both exposure history and externalizing behavior, while recognizing that the observed moderation patterns require replication. The distinction between violence witnessing and violence victimization may also be relevant, because witnessing violence could indicate greater proximity to unsafe environments without necessarily implying that direct victimization will subsequently occur.

More broadly, the present study contributes to public health research in three ways. First, it situates political violence as a population-level context relevant to adolescent health and safety rather than considering it solely as an individual trauma exposure. Second, it suggests that aggression and severe violence commission condition the longitudinal associations between terrorism exposure and community violence exposure, which is relevant to risk stratification and targeted prevention. Third, it illustrates the value of longitudinal designs for examining which adolescents may be more likely to experience both chronic threat and later violence exposure. These issues lie at the intersection of public mental health, youth violence prevention, and social epidemiology.

Although the present study was conducted among adolescents living under chronic rocket threat in southern Israel, the developmental questions it addresses may also be relevant to young people in other communities exposed to recurrent terrorism, political violence, armed conflict, or persistent security threats. Research involving Palestinian youth, children exposed to sectarian conflict, and Israeli and Palestinian adolescents has similarly shown that political violence may be associated with adjustment across multiple ecological domains, including emotional distress, aggression, family functioning, peer relations, and exposure to violence in everyday settings [17,18,19,20,21,22]. This cross-context evidence suggests that the ecological–transactional framework examined here, as well as the distinction between violence witnessing and direct victimization, may provide useful organizing concepts for research in other conflict-affected populations in the Middle East and elsewhere.

Nevertheless, the specific findings should not be assumed to generalize uniformly across conflict settings. Communities differ substantially in the nature, intensity, duration, and predictability of threat; the extent of displacement and disruption to schooling and family life; exposure to military, intergroup, or community violence; access to social and mental health services; and cultural and political context. These differences may influence both adolescents’ exposure to violence and the role of aggression or severe violence commission within their developmental environments. Accordingly, the present moderation patterns should be regarded as contextually grounded findings that generate testable hypotheses for replication in diverse conflict-exposed populations rather than as estimates that can be transferred directly to other communities.

### 4.3. Limitations and Future Directions

Several limitations should be acknowledged. First, the models explained a moderate, but not comprehensive, proportion of the variance in Time 2 community violence exposure. The analyses were designed as theory-driven tests of whether aggression and severe violence commission conditioned the associations of terrorism exposure with violence witnessing and victimization; they were not intended as exhaustive prediction models of adolescents’ later violence exposure. Important unmeasured or unmodeled influences may include affiliation with aggressive or delinquent peers, parental monitoring and family functioning, school climate and school disruption, neighborhood disorder, socioeconomic adversity, exposure to violence in family or school settings, perceived safety, social support, impulsivity, emotion regulation, and opportunities for involvement in unsafe environments. These factors were not incorporated because they were not all available within the present analytic dataset and fell outside the focal moderation hypotheses.

Their inclusion could affect the findings in several ways. Variables reflecting prior involvement in violent environments or broader contextual adversity might account for shared variance and attenuate some of the observed main or interaction effects. Other variables, such as peer affiliation, school disruption, parental monitoring, perceived safety, or emotion regulation, may function as mediating processes through which terrorism exposure becomes associated with later community violence exposure. Protective factors such as social support or effective family functioning could also introduce additional moderation effects. Consequently, the unexplained variance should not be interpreted as evidence against the observed conditional associations, but it limits their comprehensiveness and indicates that the present models capture only part of a broader developmental ecology of risk.

Relatedly, although the study employed a longitudinal design, it does not establish the mechanisms underlying the observed associations between terrorism exposure and Time 2 community violence exposure. The contextual and relational processes identified above should be tested explicitly in future longitudinal models. This issue is especially important in light of ecological–transactional theory, which proposes that broader social adversity becomes translated into developmental risk through more proximal relational and contextual processes [16]. A further temporal limitation concerns the cumulative Kassam rocket exposure measure, which incorporated exposure reports from 2008, 2009, and 2010, whereas the community violence outcomes were assessed in 2010. Consequently, temporal precedence cannot be established for the 2010 component of cumulative Kassam rocket exposure relative to the Time 2 outcomes. Associations involving this measure should therefore be interpreted as longitudinal associations across the study interval rather than as strictly prospective effects.

A second limitation concerns the study’s reliance on adolescent self-report questionnaires. Terrorism exposure, community violence exposure, aggression, severe violence commission, depressive symptoms, and anxiety symptoms were all assessed through self-report, raising the possibility of shared method variance, recall bias, and social desirability effects. Although the temporal separation between most Time 1 predictors and moderators and the Time 2 outcomes reduces the possibility that the findings reflect purely concurrent response tendencies, it does not eliminate common reporter effects. Adolescents’ tendencies to overreport or underreport threat, aggression, and violence could have influenced not only the lower-order associations but also the estimated interaction terms and conditional effects. Correlated measurement error or response-style differences across levels of aggression and severe violence commission could potentially inflate, attenuate, or alter the apparent form of the moderation patterns. This concern may be particularly relevant to cumulative Kassam rocket exposure because the 2010 exposure report and the community violence outcomes were obtained during the same assessment year. The interaction findings should therefore be regarded as provisional evidence of differential developmental processes rather than as definitive demonstrations of moderation. Future research would benefit from multi-informant designs incorporating caregiver, teacher, school, clinical, or administrative data where ethically and practically feasible.

In addition, the focal moderators may function not only as indicators of externalizing behavior but also as markers of adolescents’ prior embedding in violent social ecologies. This concern is especially relevant for severe violence commission, which may reflect both individual behavioral risk and prior involvement in unsafe environments. Relatedly, the stronger and clearer effects for witnessing than for victimization suggest that these outcomes should remain conceptually distinct in future research rather than being treated as interchangeable forms of violence exposure [14].

The study was also conducted in a highly specific geopolitical setting, namely adolescents living in the Northern Negev under chronic missile threat. That context is an important strength of the study, but it may also limit generalizability to other populations and conflict settings. In addition, because the data were collected during the 2008–2010 period, the findings should be interpreted within a historically specific context of chronic threat rather than as a timeless estimate of effect size. Finally, although Little’s test did not reject the MCAR hypothesis, locality significantly predicted missingness at the 2010 assessment. The missingness pattern may therefore be more appropriately regarded as plausibly missing at random conditional on observed locality. Locality was included as a covariate, and FIML was used to retain all available observations; nevertheless, missing data remain a methodological consideration in this longitudinal study. Future research would benefit from replication in samples with more complete follow-up data and from designs that can directly examine factors associated with continued participation over time [25].

A further limitation concerns model complexity and statistical power. Although the aggression and severe violence commission models were estimated separately, each model included multiple lower-order terms and an exploratory three-way interaction. The sample size was fixed by the available longitudinal cohort and was not selected through an a priori power analysis for detecting higher-order interactions. Interaction effects, particularly three-way interactions, generally require greater statistical power than main effects, and the restricted or skewed distributions of several variables, especially violence victimization and severe violence commission, may have further reduced the precision and stability of the estimates. In particular, Time 2 violence victimization exhibited a pronounced floor effect and highly restricted variance. Because Amos does not provide MLR or WLSMV estimation, the present maximum-likelihood models did not include a robust correction specifically designed for this distributional feature. The victimization coefficients and interaction effects should therefore be interpreted with particular caution. Restricted variability may have attenuated associations and reduced power relative to the corresponding analyses of violence witnessing. Consequently, nonsignificant interactions should not be interpreted as definitive evidence that moderation was absent. Conversely, the significant three-way interaction may be sample-specific and should be regarded as tentative until reproduced in an independent, preferably larger, conflict-exposed sample. The number of interaction tests also increases the possibility of chance findings, reinforcing the need to emphasize the overall pattern of results rather than any single conditional coefficient.

## 5. Conclusions

This study suggests that past and cumulative terrorism exposure are longitudinally associated with adolescents’ Time 2 community violence exposure, particularly violence witnessing, but that these associations depend on the form of terrorism-related exposure, the form of externalizing behavior, and the specific community-violence outcome under consideration. Across the moderation models, the clearest pattern was not one of broad stress amplification. Rather, past terror exposure was more strongly associated with later community violence exposure when aggression or severe violence commission was low, whereas these associations were attenuated, and in some cases reversed, when aggression or severe violence commission was high.

These findings support a differentiated developmental account. The ecological–transactional model provides the broader framework for understanding how chronic political violence may become linked to later exposure to unsafe social environments. At the same time, the moderation findings are more consistent with a dual-vulnerability or rival-vulnerability interpretation than with a generalized stress-diathesis model. Aggression and severe violence commission may alter the incremental predictive value of prior terrorism exposure rather than simply intensifying its effects.

From a clinical and public health perspective, the findings suggest that research and assessment concerning adolescents living under chronic terrorism or political violence may benefit from considering not only trauma-related symptoms, but also externalizing behavior and exposure to violence in everyday social environments. Distinguishing aggression from severe violence commission, and violence witnessing from direct victimization, may assist in developing more differentiated hypotheses and assessment frameworks. However, the implications for screening, prevention, and intervention remain provisional because the present study was observational, involved a geographically and historically specific cohort, and did not directly evaluate intervention processes or outcomes. Replication across diverse conflict-affected communities is therefore needed before specific practice recommendations can be advanced.

## Figures and Tables

**Table 1:** Means, variances, and standard deviations of the continuous variables.

Variable	Time	*M*	Variance	*SD*
Past terror exposure	T1	1.823	1.472	1.213
Cumulative Kassam rocket exposure	2008–2010	3.633	5.768	2.402
Depressive symptoms	T1	17.036	97.073	9.853
Anxiety symptoms	T1	2.053	0.579	0.761
Aggression	T1	1.252	1.426	1.194
Severe violence commission	T1	0.334	0.635	0.797
Violence witnessing	T1	1.461	2.544	1.595
Violence victimization	T1	0.309	0.556	0.746
Violence witnessing	T2	1.016	2.088	1.445
Violence victimization	T2	0.224	0.048	0.219

Note. T1 = 2008; T2 = 2010. Descriptive statistics are based on the FIML analyses conducted in AMOS. Ms pertain to model-implied means.

**Table 2:** Intercorrelations among model variables.

Variable	1	2	3	4	5	6	7	8	9	10	11	12
1. Past terror exposure (T1)	—											
2. Cumulative Kassam rocket exposure (2008–2010)	0.37 ***	—										
3. Depressive symptoms (T1)	0.19 **	0.12	—									
4. Anxiety symptoms (T1)	0.17 **	0.21 **	0.60 ***	—								
5. Aggression (T1)	0.27 ***	0.01	0.31 ***	0.22 **	—							
6. Severe violence commission (T1)	0.20 **	−0.07	0.15	0.05	0.53 ***	—						
7. Violence witnessing (T1)	0.20 **	0.02	0.22 **	0.23 ***	0.43 ***	0.53 ***	—					
8. Violence victimization (T1)	−0.08	−0.08	0.07	0.06	0.28 ***	0.44 ***	0.42 ***	—				
9. Violence witnessing (T2)	0.13 **	−0.02	0.15 *	0.08	0.30 ***	0.30 ***	0.45 ***	0.22 **	—			
10. Violence victimization (T2)	−0.04	0.08	0.11	0.03	0.25 ***	0.35 ***	0.15 *	0.26 **	0.37 ***	—		
11. Sex	0.08	−0.07	−0.18 **	−0.25 ***	0.20 **	0.22 **	0.13	0.18 **	0.18 **	0.18 **	—	
12. Locality	0.19 **	0.15 *	−0.05	0.05	0.00	0.11	0.17 **	0.07	0.11	−0.02	0.03	—
13. Grade	0.03	−0.01	0.18 **	0.15 *	0.04	0.05	0.10	0.07	0.09	−0.02	−0.08	0.05

Note. Values are model-implied zero-order correlations estimated using FIML with the full longitudinal study cohort (*n* = 362). Sex was coded 0 = girls, 1 = boys. Locality was coded 0 = Shaar HaNegev, 1 = Sderot. Grade reflects school grade at Time 1. * *p* < 0.05. ** *p* < 0.01. *** *p* < 0.001.

## Data Availability

Data was collected prior to the establishment of OSF. De-identified data is available from the investigators upon request.
